# Survival fluctuation is linked to precipitation variation during staging in a migratory shorebird

**DOI:** 10.1038/s41598-022-24141-5

**Published:** 2022-11-18

**Authors:** Vojtěch Brlík, Veli-Matti Pakanen, Tuomo Jaakkonen, Heikki Arppe, Jaakko Jokinen, Johanna Lakka, Donald Blomqvist, Steffen Hahn, Jari Valkama, Kari Koivula

**Affiliations:** 1 Department of Ecology, Charles University, Viničná 7, 12844 Prague, Czech Republic; 2 Czech Academy of Sciences, Institute of Vertebrate Biology, Květná 8, 60365 Brno, Czech Republic; 3 Ecology and Genetics Research Unit, University of Oulu, PO Box 3000, 90014 Oulu, Finland; 4 Department of Biological and Environmental Sciences, University of Gothenburg, Box 463, 405 30 Göteborg, Sweden; 5 Vietnam-Finland International School, Ton Duc Thang University, 01, D1 Street, District 7, Ho Chi Minh City, Vietnam; 6 Helsinki, Finland; 7 School of Forest Sciences, University of Eastern Finland, P.O. Box-111, 80101 Joensuu, Finland; 8 Department of Bird Migration, Swiss Ornithological Institute, Seerose 1, 6204 Sempach, Switzerland; 9 Finnish Museum of Natural History, University of Helsinki, P. O. Box 17, 00014 Helsinki, Finland

**Keywords:** Animal migration, Behavioural ecology, Climate-change ecology, Population dynamics

## Abstract

Understanding how weather conditions affect animal populations is essential to foresee population changes in times of global climate shifts. However, assessing year-round weather impacts on demographic parameters is hampered in migratory animals due to often unknown occurrence in space and time. We addressed this by coupling tracking and weather data to explain extensive variation in apparent survival across 19 years in a northern European population of little ringed plovers (*Charadrius dubius*). Over 90% (n = 21) of tracked individuals followed migration routes along the Indo-European flyway to south India. Building on capture–recapture histories of nearly 1400 individuals, we found that between-year variation in precipitation during post-breeding staging in northern South Asia explained 47% of variation in apparent adult survival. Overall, the intensity of the monsoon in South Asia explained 31–33% of variability in apparent survival. In contrast, weather conditions in breeding, final non-breeding and pre-breeding quarters appeared less important in this species. The integration of multi-source data seems essential for identifying key regions and periods limiting population growth, for forecasting future changes and targeting conservation efforts.

## Introduction

Weather conditions alter the fitness of animals^1–4^ hence between-year variation in weather conditions affect demographic rates of populations^5–8^. Weather conditions affect populations both during the reproductive period^3,9^ and the non-reproductive period^6,7,10–12^. Thus, a full annual perspective on these relationships is required to thoroughly understand population fluctuations^13–15^, especially, under current global climate shifts^16–18^.

Assessments of year-round relationships between weather conditions and migratory bird populations were long hampered by challenges to track movements of migratory birds^19–21^ limiting integration with weather datasets. These challenges have been overcome with recent advent in bio-logging that enable tracking of full annual cycles of migratory birds^22^. Combining population-specific tracking data, weather conditions experienced throughout the annual cycle, and long-term data on survival could help to understand the mechanisms behind the population changes in migratory species^23^. Despite the need for such knowledge, the responses of populations to year-round weather conditions are unknown in many terrestrial birds^8,12,24^.

**Figure 1:**
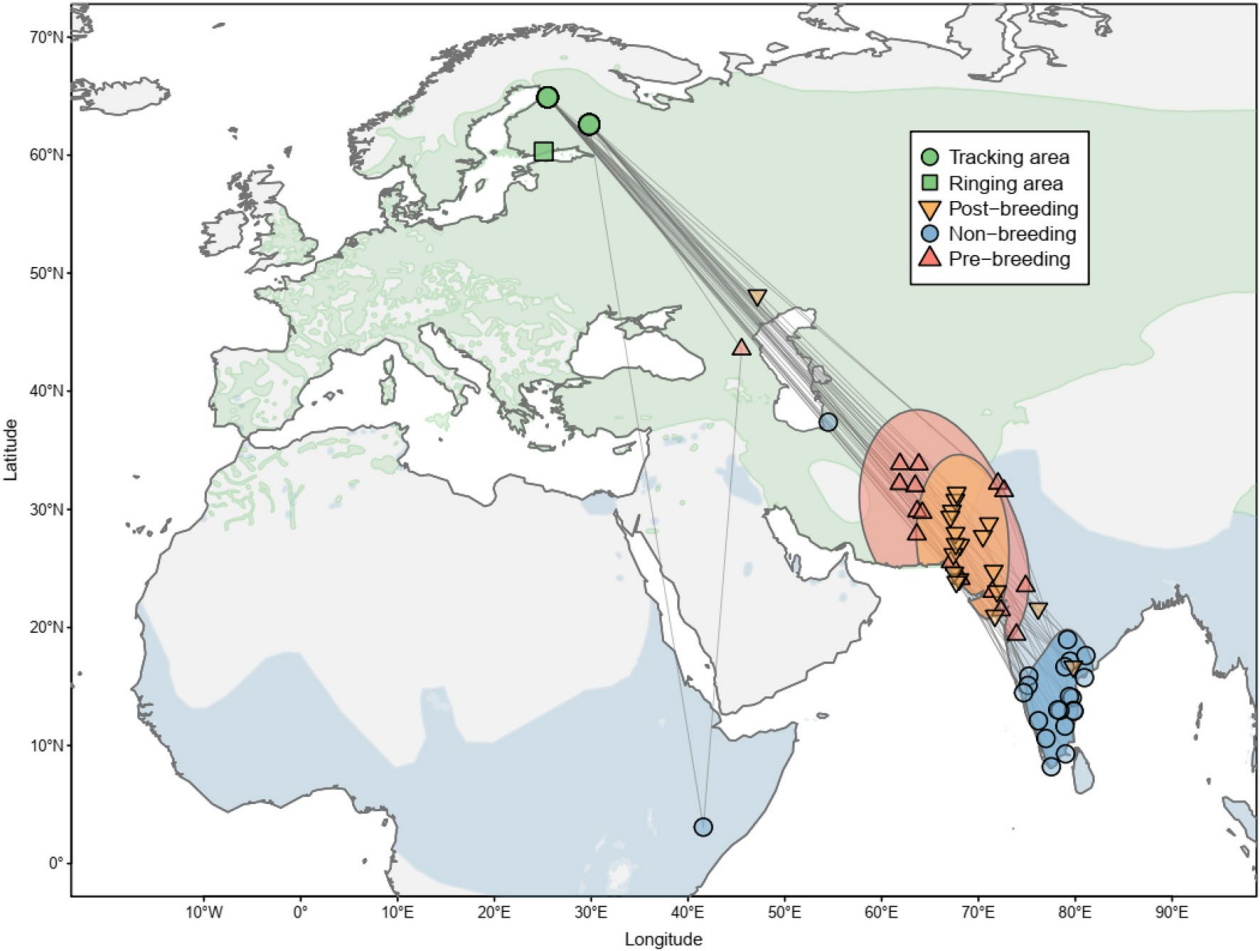
Distribution range of the little ringed plover (*Charadrius dubius*; light-coloured polygons^27^) and the main regions (kernel density estimates; polygons) where tracked individuals (n = 21; dots and triangles) occurred during the main annual cycle periods (Table 1). Coordinates of the geographic positions (dots and triangles) represent modus of daily positions derived from geolocators. Lines connect geographic positions and do not reflect migratory routes.

Here, we use full annual tracking to acquire detailed weather conditions experienced by little ringed plovers (*Charadrius dubius;* Fig. 1) from a north European breeding population and link these conditions with demographic data. The little ringed plover is a small, long-distance migratory shorebird breeding across the Palearctic^25^. The European breeding populations can use both the Afro-Palearctic and the Indo-European migratory flyways^26,27^ but the population-specific migratory patterns are mostly unknown. Importantly, apparent adult survival varies largely between years in a north European little ringed plover population, but the source of variation remains unknown^28^. Building on the current knowledge of migratory patterns in the species and relationships between weather conditions and migratory populations, we predict the following:

1. Population-specific tracking data will enable detailed assessment of the weather–apparent survival relationships despite highly variable and currently largely unknown migratory patterns in the little ringed plover^26,27^.
2. Weather conditions (precipitation and temperature) will affect apparent survival of the little ringed plovers because they affect the availability of ephemeral wetland habitats and food abundance^29,30^.
3. Weather conditions outside breeding grounds will play a crucial role for the apparent survival of the little ringed plover breeding in northern Europe^31,32^.

## Results

### Migratory pattern

Majority (19 out of 21) of the tracked little ringed plovers followed the Indo-European migratory flyway from Northern Europe to South Asia (Fig. 1). The migratory patterns were similar in these individuals that spent 53 ± 29 days (mean ± SD, n = 19) in the post-breeding staging region in northern South Asia, 164 ± 30 days (n = 15) in the non-breeding region in south India, and 16 ± 6 days (n = 15) in the pre-breeding staging region in northern South Asia (Fig. 1, Table 1). Beside these 19 individuals, one individual spent the main non-breeding period (236 days) close to the Caspian Sea without longer stopovers (> 6 days) during migration. A second individual followed the Afro-Palearctic migratory flyway through a post-breeding staging site in central Middle East (77 days) to East Africa (151 days) with a short stop (< 6 days) close to the Caspian Sea during northward migration (Fig. 1). We detected no statistically significant differences in the duration of the main annual cycle periods between females and males: the average differences (males–females) for post-, non- and pre-breeding periods were 0.5, 1.1 and − 0.6 days, respectively (Mann–Whitney non-parametric test P values 0.90, 0.68 and 0.95).

**Table 1:** Timing of the main annual cycle periods of little ringed plovers (*Charadrius dubius*) breeding in north Europe derived from tracking data (see “Material and methods” for details).

Period	Arrival	N	Departure	N
Post-breeding staging	14 August	19	6 October	19
Non-breeding	12 October	19	23 March	15
Pre-breeding staging	27 March	15	13 April	15
Breeding	1 April	–	30 April	–

The breeding period reflects a period around the mean arrival date at the breeding site estimated from tracking data (18 April, n = 14).

### Between-year variation in weather conditions

**Figure 2:**
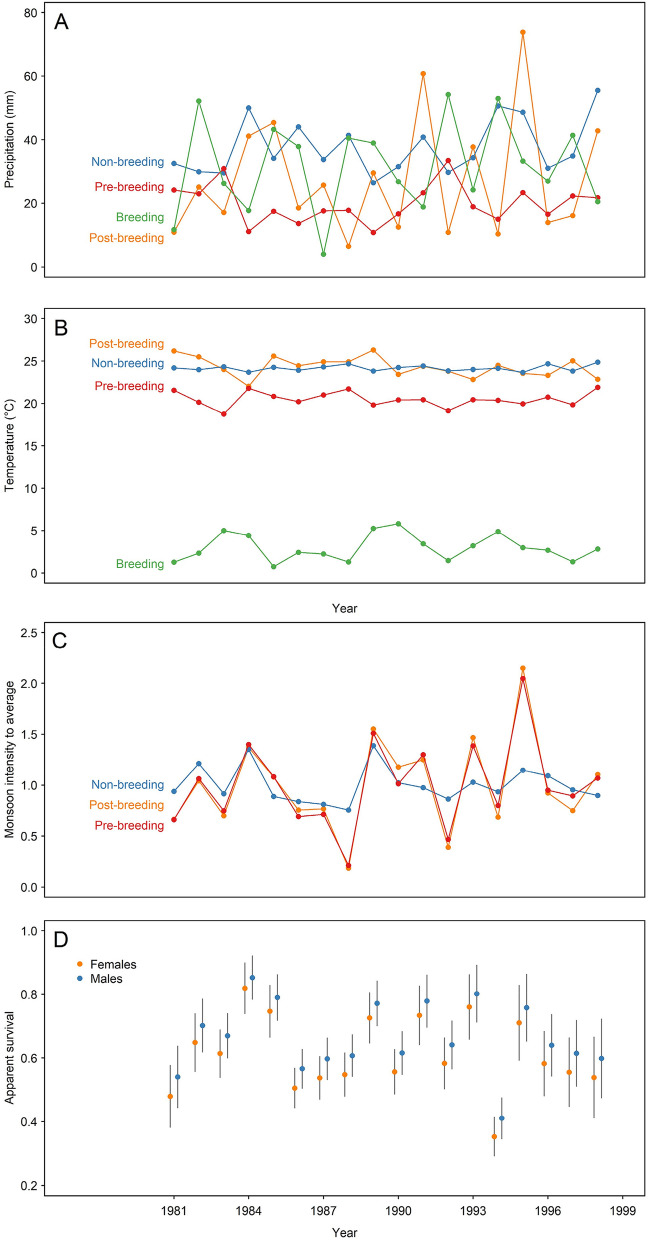
Between-year variation in amount of precipitation (**A**) and near-surface mean air temperature (**B**) during four main annual cycle periods of little ringed plovers (*Charadrius dubius*). (**C**) Between-year variation in the amount of precipitation during monsoon period (July–September; relative values presented). (**D**) Between-year variation in apparent survival estimates of adult little ringed plovers (mean ± SE) estimated from the time dependent model (Table 2).

The amount of precipitation varied largely between years in the four key regions visited by the tracked birds (coefficients of variation [CV] = 0.23–0.69, n = 18 for each region) and the temporal pattern differed between these regions (absolute Spearman correlation coefficients [|ρ|] ≤ 0.35; Fig. 2A). The pattern of between-year changes in near-surface mean air temperature differed between regions (|ρ|≤ 0.47), and the variation was extensive only in the breeding region (CV = 0.52; mean = 3 °C) and showed low variation outside breeding region with CV < 0.05 and mean > 20 °C (Fig. 2B). In contrast, the patterns of between-year changes in precipitation during the monsoon period showed strong correlation (|ρ|= 0.57–0.95) and high variation in the three non-breeding regions (CV = 0.18–0.46; Fig. 2C).

### Predictors of the apparent survival fluctuation

**Figure 3:**
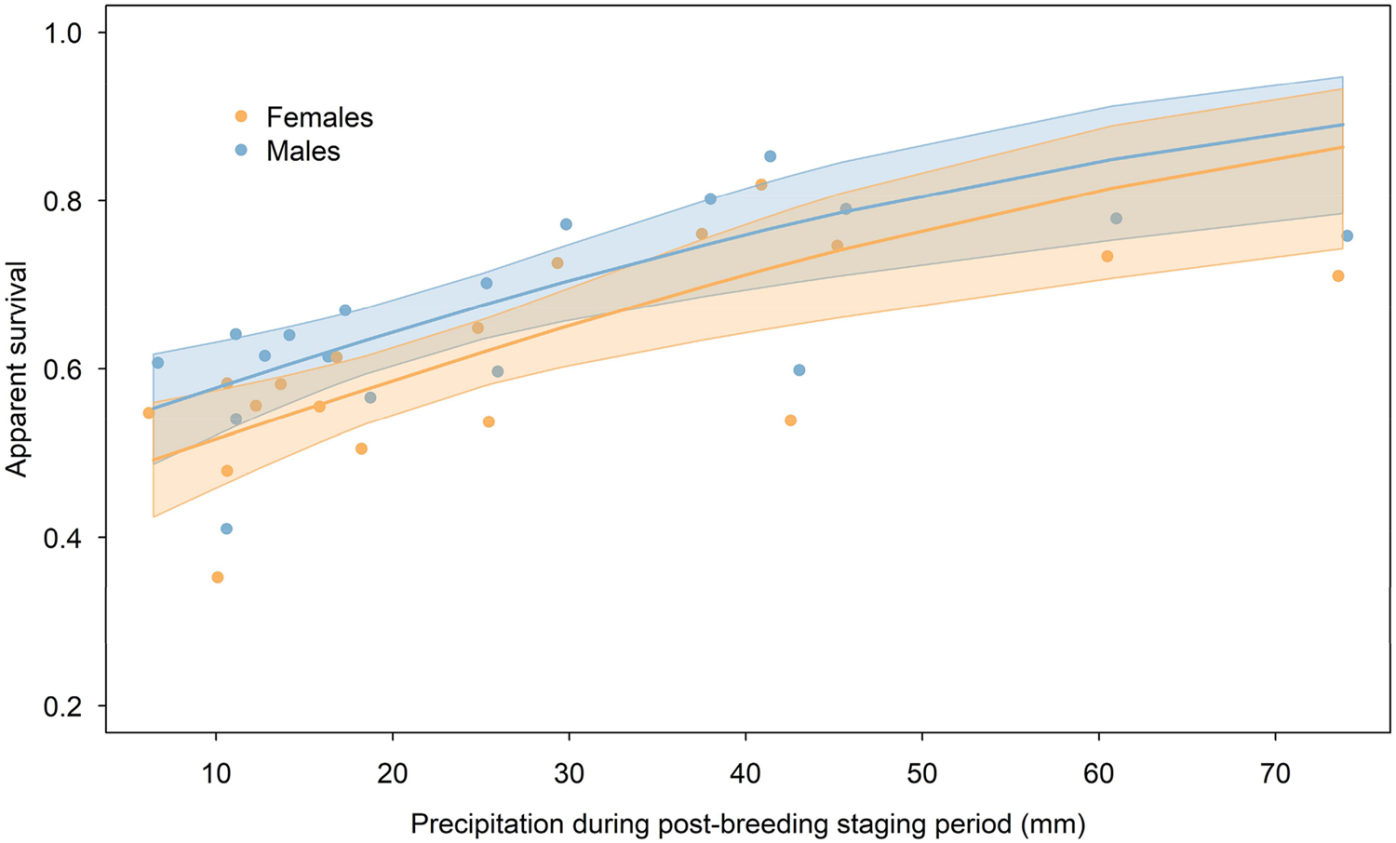
Apparent survival (with 95% confidence intervals) of little ringed plovers (*Charadrius dubius*) in relation to precipitation in the post-breeding staging region in South Asia (model ‘Precipitation post-breeding’ from Table 2). The annual estimates from the time dependent model are shown as dots.

Apparent survival of the little ringed plovers varied substantially between years (ɸ = 0.35–0.85; ΔAICc = 6.04 compared to constant model, Table 2; Fig. 2D) and males exhibited higher survival than females, as previously found in ^28^ (Fig. 2D; Table 2). High temporal variation in annual survival was best explained (47% explained variation) by a positive relationship with amount of precipitation in the post-breeding staging region in northern South Asia (ΔAICc = 17.14 compared to constant model; Fig. 3; Table 2). Overall, the amount of precipitation during the monsoon period (July–September) in the staging regions (pre-breeding: ΔAICc = 11.17 and post-breeding ΔAICc = 10.76) and in the non-breeding region (ΔAICc = 10.46) received less support compared to the best model but remained more supported than the constant model and explained 31–33% of the variation in apparent survival (Table 2). Near-surface mean air temperature in the post-breeding and non-breeding regions was only weakly linked to survival and explained 8% and 11% of temporal variability in apparent survival (ΔAICc = 1.15 and 2.32; Table 2). Similarly, the amount of precipitation during the non-breeding, pre-breeding and breeding periods explained only little variation in apparent survival, as was the case also for near-surface mean air temperature in the pre-breeding and breeding regions (Table 2).

**Table 2:** Models explaining temporal variation in apparent adult survival of little ringed plovers (*Charadrius dubius*) with weather variables.

Model	AICc	ΔAICc	w	k	Deviance	%Exp	β	SE
Recipitation post-breeding	4100.56	0.00	0.883	6	4088.52	47	0.0279	0.0075
Monsoon pre-breeding	4106.53	5.97	0.045	6	4094.49	33	0.2404	0.0947
Monsoon post-breeding	4106.94	6.38	0.036	6	4094.90	32	0.0157	0.0046
Monsoon non-breeding	4107.24	6.68	0.031	6	4095.20	31	0.0062	0.0019
Time dependent	4111.67	11.11	0.003	22	4067.16			
Temperature non-breeding	4115.38	14.82	0.001	6	4103.34	11	− 0.5473	0.2576
Temperature post-breeding	4116.55	15.99	0.000	6	4104.51	8	− 0.1081	0.0615
Constant	4117.70	17.14	0.000	5	4107.67			
Temperature breeding	4118.32	17.76	0.000	6	4106.28	3	0.0546	0.0466
Precipitation breeding	4119.11	18.55	0.000	6	4107.07	1	− 0.0052	0.0068
Precipitation non-breeding	4119.13	18.57	0.000	6	4107.08	1	− 0.0074	0.0095
Precipitation pre-breeding	4119.66	19.10	0.000	6	4107.62	0	− 0.0025	0.0110
Temperature pre-breeding	4119.71	19.15	0.000	6	4107.67	0	0.0017	0.0911

We extracted precipitation (Prec) and temperature (Temp) variables for the post-breeding staging, non-breeding, pre-breeding staging, and breeding periods in corresponding regions (see Table 1 and Fig. 1 for details). The monsoon variables were extracted for the same regions for July–September. The underlying model structure included always sex for survival and trap dependence (m) and linear trend (trend) for recapture probabilities, i.e., Φ(sex) p(m + trend). AICc = Akaike’s information criterion corrected for small sample size; *ΔAICc* AICc difference from the best supported model, *w* Akaike weight, *k* number of parameters, *%Exp* percentage of temporal variation in survival explained by the covariate, *β* coefficient in logit scale, *SE* standard error of coefficient.

## Discussion

We show that the fluctuation in adult survival of a long-distance migratory species is associated with cross-continental weather conditions. By tracking, we uncovered the spatiotemporal distribution of the little ringed plover breeding in northern Europe, which was essential to narrow down the regions and periods of importance. By combining this information with weather and long-term breeding capture-recapture data, we pinpointed the weather conditions experienced throughout the annual cycle that are linked to high temporal variation in apparent survival. In particular, the amount of precipitation during the post-breeding staging period explained 47% of the temporal variation in survival. Our results show that the conditions during migratory periods and staging are important and have repercussions for individuals^33,34^ and illustrate the value of a full-annual perspective in such studies to provide essential knowledge on cross-continental relationships between weather conditions and survival rates.

The apparent survival of little ringed plovers breeding in northern Europe showed a strong positive link to precipitation during multiple non-breeding periods in South Asia. These findings are consistent with previous findings on positive relationships between survival and precipitation during the staging and other non-breeding periods^10,34,35^. Little ringed plovers migrated from Finnish breeding grounds to South Asian non-breeding quarters by crossing the vast Palearctic landmasses. Therefore, rains during the post-breeding period largely coinciding with the Asian summer monsoon form the only major climatic event they experience, which may explain the strong link between this event and their annual survival.

While multiple studies have shown negative impacts of low temperatures on survival of short-distance migratory shorebirds^36–38^, our study is among the first to show an effect of precipitation on adult survival in a long-distance migratory shorebird. Previously, a link between adult survival of common sandpipers (*Actitis hypoleuca*) and the North Atlantic Oscillation was detected^39^, indicating that warm and wet conditions in the wintering areas result in higher survival but that precipitation has no effect on survival. However, we detected only very little between-year variation in temperature measures at the non-breeding sites far below the diurnal and annual changes experienced by the birds. Our results are consistent with studies suggesting that long-distance migratory shorebirds mainly incur mortality at the non-breeding sites^31,40^. The lack of previous studies reporting a relationship between precipitation and survival of long-distance migratory shorebirds may reflect the fact that shorebirds generally exhibit low levels of temporal variation in adult survival and high survival compared to other similar-sized birds^41–43^. Interestingly, plovers (genus *Charadrius*) have lower survival than expected from their body size^43^ and exhibit substantial annual variation in survival (e.g.,^44^ and this study). Pronounced annual variation in survival suggests that they are more vulnerable to environmental conditions than other shorebird species.

Susceptibility to environmental conditions may be linked to variation in the quality of non-breeding habitats. Similar to other long-distance shorebirds that migrate across continents and opportunistically stop at suitable sites^45^, the little ringed plovers are known to use staging sites such as sandbanks, rivers and lakes, rice fields, residual flood waters, short-vegetated areas near villages or water, airfields, and pastures^30,46^. Some of these habitats are highly dependent on water level and may thus make little ringed plovers susceptible to drying of the foraging habitats. Dry conditions affect the presence of water bodies, which reduces availability of invertebrate food in the staging areas^47^. Hence, drying of the staging habitats may have severe consequences for refueling performance, body condition, success of migration^48–50^, and ultimately survival, as documented here.

Precipitation during the post-breeding staging was more important than during the pre-breeding and non-breeding periods. Dry conditions during post-breeding staging may be more critical for survival because post-breeding staging occurs after an energetically-demanding migratory period^51^ and is accompanied by the moult of flight feathers that occurs mainly after August^52^. Importantly, it appears that there is a relatively strong impact of the summer monsoon (but see model supports) extending to the entire non-breeding period of the little ringed plovers and potentially affecting their survival throughout this period of the annual cycle. As the magnitude and direction of climatic changes differ between regions^53^, our results can be used to assess potential future impacts because the amount of precipitation is predicted to increase in South Asia^54^. This may benefit some long-distance migrants such as the little ringed plover via increased survival. However, marked temporal variation in weather conditions may also increase variation in survival potentially translating to population growth rates and consequently depress population size in the long-term (e.g.,^8^).

Most of the tracked little ringed plovers followed the Indo-European migratory flyway that has been previously described mainly for passerines (see^55–57^). Such a low spread within the non-breeding grounds was unexpected as European ringing recoveries show rather large variation in non-breeding directions and sites^27,58–60^ and contrasts with the Swedish little ringed plovers that cover almost an entire nonbreeding range of the species^26^. A potential limitation to our results is a spatiotemporal mismatch between survival and tracking datasets. However, we do not expect extensive mixing of the studied population with a population from southern Sweden with highly variable migratory patterns^26^ because of short natal and breeding dispersal distances^28^, and the presence of the Bothnian Bay likely forming a barrier between these populations. This explanation is also indirectly supported by the ring recoveries^59^.

In our study, the amount of precipitation explained up to 47% of the temporal variation in apparent survival of adults, more than reported in previous studies on terrestrial birds (< 18%; e.g.,^15,61,62^). Such a strong association likely reflects a precise match between the tracking and weather datasets advocating and further supporting our assumption that the breeding population largely follows the migration schedule and pattern of the tracked individuals.

Weather conditions in the staging region during post-breeding migration appears to limit survival in the population of this migratory shorebird and considerable amount of explained variation suggests susceptibility of the population to the weather conditions in this short period of the annual cycle. Our results pinpointed the region limiting the population for which the climatic models forecast long-term increase in the amount of precipitation that might prevent population declines in the species. We believe that long-term studies of populations and integration of their migratory patterns with environmental conditions will become a crucial step for better understanding large-scale population size dynamics, forecasting future changes and targeting conservation efforts in migratory birds.

## Material and methods

We studied a north European breeding population of little ringed plovers to assess the potential impacts of between-year changes in weather conditions on adult survival. First, we gathered tracking data from two ‘tracking’ areas (62° 36′ N, 29° 48′ E and 65° 00′ N, 25° 30′ E) to identify migratory pattern in the north European population (Fig. 1). Second, we estimated apparent survival of adult individuals from data collected in a ‘ringing’ area (60° 17′ N, 25° 6′ E; Fig. 1).

### Tagging and individual spatiotemporal distribution

We tagged 91 breeding adults (39 females, 43 males, 9 unidentified) in two tracking areas during 2015 and 2016 with light-level geolocators to determine their spatiotemporal distribution throughout the annual cycle. The mean body mass of tagged individuals was 38.3 ± 2.4 g (SD; n = 74), corresponding to relative tag load of 2.3% (mean; range 1.8; 3.3%), and we assume negligible effects of tagging on obtained migratory behaviour^63,64^. In the years following tag deployment, we recovered tags with at least 6 months of ambient light recording from 21 individuals (Electronic Supplementary Material 1).

We determined the geographic locations outside the breeding areas and the timing of annual cycle events using the ambient light intensity recordings from the geolocators. We estimated times of sunrises and sunsets from the log-transformed data (twGeos R package)^65^; and for further analysis used functions from GeoLight package version 2.0.0^66^. We identified stationary periods (changeLight function; quantile = 0.9; days = 2) and calculated daily geographic positions using sun elevation angles (SEA) derived by Hill-Ekstrom calibration on the longest non-breeding stationary period^67^. We merged consecutive periods with overlapping locations and applied the Hill-Ekstrom calibration to derive final SEAs (mean = −6.7°; SD = 1.3, n = 21). In two individuals, the final SEA resulted in locations in the ocean, and we thus adjusted the SEAs (by −0.5° and −0.9°) to move the locations to the nearest land.

### Population spatiotemporal distribution

Out of 21 tracked individuals, 19 spent the non-breeding period in southern India (Fig. 1). Only one individual spent the non-breeding period in Iran and one individual in Africa (Fig. 1) preventing us to robustly specify their space and time use. Hence, we considered the spatiotemporal distribution of 19 individuals following migratory routes to South Asia as the prevalent migratory strategy in the studied population (more details in “Discussion”).

To describe the population-specific space use outside the breeding areas, we extracted individual timing and daily position estimates during the following main periods: (1) the longest stationary period during southward migration (hereafter ‘post-breeding staging’), (2) the stationary period when the individual was farthest from the tracking areas (hereafter ‘non-breeding’), and (3) the longest stationary period during the northward migration (hereafter ‘pre-breeding staging’). The individual migration schedules are presented in Electronic Supplementary Material 2. We calculated the population timing of migration as the average of the first (arrivals) and the last (departures) days of the main periods (Table 1). In addition, we considered ‘breeding period’ (1–30 April) as the period around breeding site arrival in the tracked birds (mean = 18 April; n = 14). Sample sizes for timing estimates differ between main periods due to premature battery failures in some geolocators (Table 1). The breeding region is defined as the area surrounding the ringing area (described below; 60° 08′–60° 35′ N; 24° 49′–25° 49′E). We estimated the population space use outside the breeding areas during the main periods (hereafter ‘main regions’ together with the breeding region) from bi-daily position estimates using kernel density estimates. We applied kde.points function (GISTools R package)^68^ with a bandwidth of 10, and omitted the lowest 2% of density estimates and clipped out areas over seawater (Fig. 1).

### Weather conditions during the annual cycle

In total, we extracted four precipitation and four near-surface mean air temperature measures matching spatiotemporal distribution, and three monsoon measures matching only spatial distribution. Weather variables were extracted from TerraClimate^69^ using the spatiotemporal distribution of the tracked population (Fig. 1; Table 1) by calculating weighted average of monthly values using the number of days in each month as a weight. We obtained these measures for seasons preceding the breeding periods 1981–1998 (see below). In addition, we collected information on the amount of precipitation in three main regions outside breeding areas (Fig. 1) during summer monsoon period in July–September; a major climatic event in South Asia^70^. We used R version 4.1.2 for the analysis^71^ and Google Earth Engine to gather weather data^72^.

### Capture–recapture and apparent survival analysis

We used capture–recapture histories of 1386 individuals (747 females, 639 males) collected in the ringing area (Fig. 1) to estimate apparent survival of the little ringed plover in 1980–1998. The dataset is detailed in^28^ and summarised in Electronic Supplementary Material 3. We employed Cormack-Jolly-Seber (CJS) models adapted for open populations^73^ in program MARK version 9.0^74^. Our initial model included sex, time (t) and their interaction (*) for survival (Φ), and recapture probabilities (p) included also immediate trap-dependence (m; see^75^) on capture probabilities [Φ(sex*t) p(sex*m*t)]. We assessed goodness-of-fit with U-CARE 2.3.2^76^ and found that this model fit the data^28^. In this study, we continue with the best-fit structure from^28^, i.e. [Φ(sex + t) p(sex + trend)] which includes sex and time for survival and trap response and temporal trend for recapture probabilities. In other models, we only varied the structure for time dependence in survival. In addition to the starting model in which survival was time dependent, we fit models where survival was constant in time or constrained by one of the 11 weather variables. Sex was included for survival in all models because females were found to have lower apparent survival than males in the previous study^28^. This sex difference in survival is likely caused by differences in permanent emigration as females disperse further than males^28^. This potential permanent emigration can reduce the apparent survival estimates. As breeding dispersal decisions are mostly linked to breeding success in shorebirds (e.g.,^77^) and the changing quality of the ephemeral breeding sites (mostly man-made habitats^28^) of the little ringed plover in this study population, it is not likely that permanent emigration would be linked to the weather conditions experienced in the non-breeding sites.

We compared models using the Akaike information criterion adjusted for small sample size (AICc)^78^ and considered a difference of at least 2 AICc units to infer a difference in model support. We assessed support for time dependence and weather variables by comparing those models to the constant model. In addition, we examined the percentage of temporal variation explained by the weather covariates by comparing deviances the weather covariate models to constant and time dependent models. We calculated the percentage of deviance explained by the covariate model following^79^ as

$$(\mathrm{Dev}(\mathrm{c})-\mathrm{Dev}(\mathrm{cov})) / (\mathrm{Dev}(\mathrm{c})-\mathrm{Dev}(\mathrm{t})),$$

where Dev(c) is the deviance from the constant model, Dev(cov) is deviance from the covariate model and Dev(t) is the deviance from the time dependent model.

### Ethical approval

This study complied with national law and the reporting in the manuscript follows the recommendations in the ARRIVE guidelines. The experimental protocols and methods were approved by the Centre for Economic Development, Transport and the Environment in Finland (permit number VARELY/1088/2015) and the Ringing Centre of the Finnish Museum of Natural History. All methods were carried out in accordance with relevant guidelines and regulations.

## Supplementary Information

Supplementary Information.

## Data Availability

Data are available from Zenodo data repository^80^ and MoveBank (ID 2279403362).
