# Supplementary material for: Survival fluctuation is linked to precipitation variation during staging in a migratory shorebird
Source: Sci Rep. 2022 Nov 18;12:19830. doi: 10.1038/s41598-022-24141-5 (PMC9674593; doi:10.1038/s41598-022-24141-5)
Supplement: Supplementary file 1 — Supplementary Information. [file 41598_2022_24141_MOESM1_ESM.pdf]

Survival fluctuation is linked to precipitation variation during staging in a migratory shorebird

Vojtěch Brlík[1,2,*], Veli-Matti Pakanen[3,4,*], Tuomo Jaakkonen[3,5], Heikki Arppe[6], Jaakko Jokinen[3], Johanna Lakka[7], Donald Blomqvist[4], Steffen Hahn[8], Jari Valkama[9], Kari Koivula[3]

[*] contributed equally

[1] Department of Ecology, Charles University, Viničná 7, 12844 Prague, Czech Republic

[2] Czech Academy of Sciences, Institute of Vertebrate Biology, Květná 8, 60365 Brno, Czech Republic

[3] Ecology and Genetics Research unit, University of Oulu, PO Box 3000, 90014 Oulu, Finland

[4] Department of Biological and Environmental Sciences, University of Gothenburg, Box 463, Gothenburg, 405 30, Sweden

[5] Vietnam-Finland International School, Ton Duc Thang University, 01, D1 Street, District 7, Ho Chi Minh City, Vietnam

[6] Deceased

[7] University of Eastern Finland, School of Forest Sciences, P.O. Box-111, FI-80101 Joensuu, Finland

[8] Department of Bird Migration, Swiss Ornithological Institute, Seerose 1, 6204 Sempach, Switzerland

[9] Finnish Museum of Natural History, P. O. Box 17, 00014 University of Helsinki, Finland

Corresponding authors: Vojtěch Brlík (vojtech.brlik@gmail.com); Veli-Matti Pakanen (veli-matti.pakanen@oulu.fi)

Electronic Supplementary Material

Electronic Supplementary Material 1

Details of the geolocator types and mass (including harness or plastic flag), type of attachment, and number of devices deployed and recaptured.

Geolocator type	*Mass (g)*	*Attachment*	*Deployed*	*Recaptured*
MigTech C65	1.15	Leg flag	8	1
MigTech P50B1-7	0.70	Leg-loop harness	7	2
MigTech P65-A22-11	0.85	Leg-loop harness	19	4
Swiss Ornithological Institute GDL2	0.75	Leg-loop harness	9	3
MigTech W50A9	0.85	Leg flag	18	3
MigTech W65A9	0.85	Leg flag	30	8

Electronic Supplementary Material 2

Individual migratory schedules for the little ringed plovers (*Charadrius dubius*) breeding in northern Europe derived from light-level geolocators. The boxes represent stationary periods (green – breeding; orange – post-breeding; blue – non-breeding; red – pre-breeding). The migratory destinations are divided according to the three main migratory destinations detected (Africa, South Asia, the Caspian Sea). The individuals are sorted according to their migratory destination, sex and the length of the main non-breeding period.

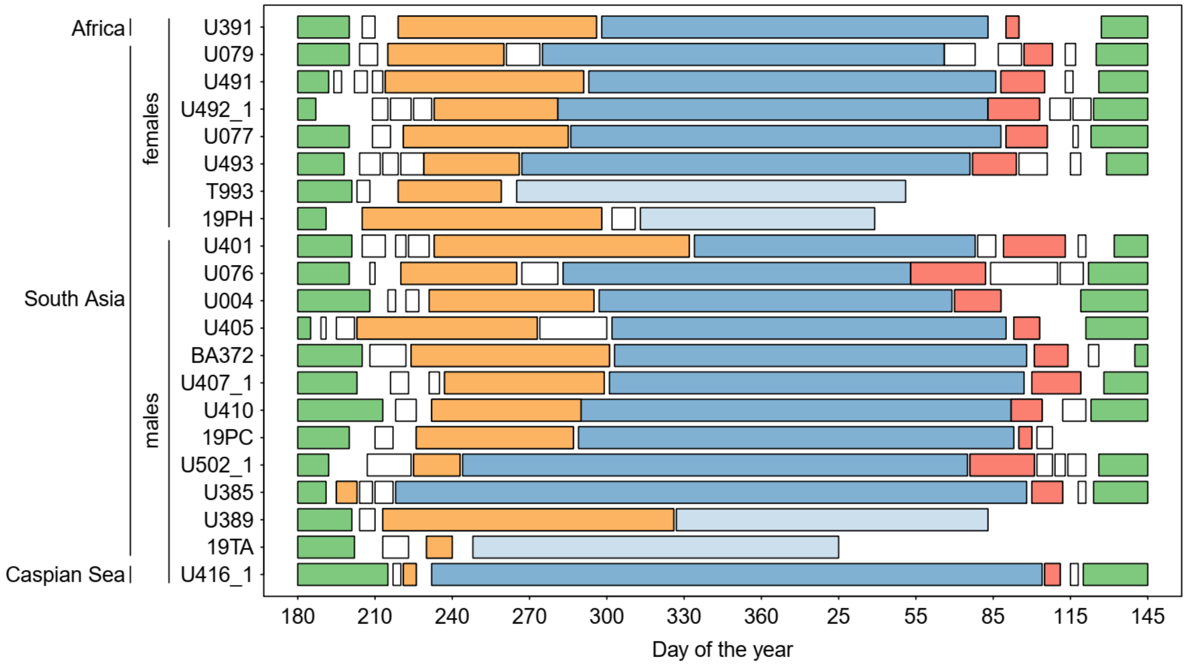

Electronic Supplementary Material 3

Number of females and males that were observed each year. This includes both the newly marked individuals (1386 in total) and their subsequent recaptures.

	1980	1981	1982	1983	1984	1985	1986	1987	1988	1989	1990	1991	1992	1993	1994	1995	1996	1997	1998	Sum
Males	32	39	45	66	67	78	66	68	81	54	52	53	59	55	37	44	38	36	30	1000
Females	33	50	54	79	73	89	78	59	73	69	56	56	66	59	35	55	47	42	32	1105
Both	65	89	99	145	140	167	144	127	154	123	108	109	125	114	72	99	85	78	62	2105
